# Case-mix & patients' reports of outcome in Independent Sector Treatment Centres: Comparison with NHS providers

**DOI:** 10.1186/1472-6963-8-78

**Published:** 2008-04-09

**Authors:** John Browne, Liz Jamieson, Jim Lewsey, Jan van der Meulen, Lynn Copley, Nick Black

**Affiliations:** 1 Health Services Research Unit, London School of Hygiene & Tropical Medicine, UK; 2 Clinical Effectiveness Unit, Royal College of Surgeons, UK

## Abstract

**Background:**

There has been considerable concern expressed about the outcomes achieved in Independent Sector Treatment Centres (ISTCs) introduced in England since 2003. Our aim was to compare the case-mix and patients' reported outcomes of surgery in ISTCs and in NHS providers.

**Methods:**

Prospective cohort study of 769 patients treated in six ISTCs and 1895 treated in 20 NHS providers (acute hospitals and treatment centres) in England during 2006–07. Participants underwent one of three day surgery procedures (inguinal hernia repair, varicose vein surgery, cataract extraction) or hip or knee replacement. Change in patient-reported health status and health related quality of life (measured using a disease-specific and a generic (EQ-5D) instrument) was assessed either 3-months (day surgery) or 6-months (hip/knee) after surgery. In addition patient-reported post-operative complications and an overall assessment of success of surgery were collected. Outcome measures were adjusted (using multivariable regression) for patient characteristics (disease severity, duration of symptoms, age, sex, socioeconomic status, general health, previous similar surgery, comorbidity).

**Results:**

Post-operative response rates varied by procedure (73%–88%) and were similar for those treated in ISTCs and NHS facilities. Patients treated in ISTCs were healthier, were less likely to have any comorbidity and, for those undergoing cataract surgery or joint replacement, their primary condition was less severe. Those undergoing hernia repair or joint replacement were less likely to have had similar surgery before.

When adjustment was made for pre-operative characteristics, patients undergoing cataract surgery or hip replacement in ISTCs achieved a slightly greater improvement in functional status and quality of life than those treated in NHS facilities, while the opposite was true of patients undergoing hernia repair. No significant differences were found for the two other procedures. Patients treated in ISTCs were less likely to report post-operative problems than those treated in NHS facilities for cataract surgery (Adjusted Odds Ratio 0.35; 95% CI 0.17–0.70), hernia repair (0.42; 0.28–0.63) and knee replacement (0.44; 0.28–0.69). Most patients described the result of their operation as excellent, very good or good, regardless of where they were treated.

**Conclusion:**

The case-mix of patients treated in ISTCs differs from that in NHS providers, in line with the intention of the contracts. Caution is needed in interpreting the observation that patients treated in ISTCs reported slightly better outcomes as very few ISTCs participated, case-mix adjustment might have been insufficient, and patients' reports might have been biased as they were more likely to be satisfied with the way they were treated.

## Background

In 2002 the Department of Health committed to increasing the permanent capacity for elective surgery (and diagnostic services) in England by establishing more Diagnosis and Treatment Centres [1]. The aim was not only to reduce waiting times for routine procedures but also to expand choice and to increase productivity through innovative models of care. Unlike the ten existing NHS centres (including one run jointly with a private provider), expansion was partly to be achieved by encouraging private companies to establish facilities employing staff not currently working for the NHS, thus creating additional capacity in England. The first of these Independent Sector Treatment Centres (ISTCs), focusing on low risk ophthalmic, orthopaedic and day surgery, opened in 2003. By October 2004, six centres had been established and had treated 16 000 patients [2].

Following some concerns about the policy of introducing ISTCs [3,4], the House of Commons Health Committee undertook a review in spring 2006. In addition to receiving evidence about poor value for money, adverse impact on surgical training, loss of continuity of care, lack of adequate clinical governance, and jeopardising the viability of existing NHS providers, clinicians reported seeing serious complications in patients who had been treated in ISTCs [5-8]. However, as some of them acknowledged, meaningful comparisons with NHS providers could not be made both because of the poor quality of the data collected by ISTCs [9] and the historical lack of routine data on outcome collected by the NHS. When the Health Committee reported in July 2006, it agreed "that there was no hard, quantifiable evidence to prove that standards in ISTCs differed from those in the NHS" and "recommended that comparable and standardised data be collected" [10]. This was welcomed by clinicians [11], some of whom had been advocating such an approach [12].

Meanwhile, in April 2006 the Secretary of State for Health pre-empted the Health Committee's report by announcing the establishment of "a wide-ranging clinical audit" to be carried out by the Healthcare Commission [13]. However, inevitably, when it appeared in July 2007, it too confirmed the lack of available routine data with which to assess outcomes [14].

Fortunately, in 2004 the Department of Health's Economic and Operational Research Division foresaw the need for meaningful comparisons of the performance of providers in the emerging world of choice. They commissioned a project to review international evidence on patient-reported outcome measures for five elective surgical procedures and to establish a demonstration project with three objectives: to test the feasibility of data collection in ISTCs and NHS providers; to establish how best to analyse the data; and to explore how best to present inter-provider comparisons. As only two ISTCs agreed to take part, the DH Commercial Directorate subsequently funded data collection from an additional four centres. These combined data provide the first opportunity to address some of the concerns of clinicians, the Health Committee, independent sector providers and policy-makers.

In this paper our objectives are to compare the case-mix of ISTCs and NHS providers (acute hospitals and treatment centres) and patient-reported outcomes in terms of change in health status/quality of life, post-operative complications and overall result of surgery. Comparisons of individual centres will appear elsewhere.

## Methods

Twenty-six providers (13 NHS hospitals, 7 NHS treatment centres, 6 ISTCs) participated. Ethical approval was granted by the Wales MREC. Consecutive patients (apart from those with cognitive impairment, poor sight, literacy or language comprehension problems) were eligible. Recruitment, which lasted for up to six months between January 2006 and April 2007, was carried out by nursing or administrative staff who explained what the study involved (completion of a pre and post-operative questionnaire) and obtained written consent. This took place either in a pre-operative assessment clinic or on admission for surgery. The pre-operative questionnaire that included: age, sex and postcode (to determine an Index of Multiple Deprivation [15] – higher scores indicate more deprived); duration of symptoms; history of previous similar surgery; general health status (five categories); co-morbidities [16]; and the EQ-5D, a generic measure of health-related quality of life [17]. In addition, a disease-specific measure of functional status/health-related quality of life was included: the VF14 for cataract surgery [18]; the Aberdeen Varicose Vein Questionnaire – AVVQ [19]; the Oxford Hip Score – OHS [20]; and the Oxford Knee Score – OKS [21]. As no specific measure for hernia repair has been validated, the Physical Component Summary (PCS) of the SF-36 was used [22]. Higher scores represent better outcomes for the EQ-5D, the PCS and the VF14. The reverse is true for the OHS, OKS and the AVVQ.

Post-operative questionnaires were mailed three (for hernia, cataract and varicose vein surgery) or six months (joint replacement) after surgery to patients' homes from the Royal College of Surgeons of England. Non-responders were sent a reminder letter and replacement questionnaire five weeks after the original mailing. The questionnaires contained: general health status; EQ-5D; a relevant disease-specific instrument; a question relating to complications – "Did you experience any of the following problems after your operation?" (allergy or reaction to drug, and urinary, bleeding or wound problems) [23]; and a global evaluation – "How would you describe the results of your operation?".

Summary statistics of pre-operative characteristics are reported for each operation group by treatment sector. Post-operative response bias was investigated by using t-tests to compare the pre-operative disease-specific instrument scores of patients who did and did not respond. The primary outcomes were the post-operative generic and disease-specific PROM scores, adjusted for pre-operative patient characteristics (age, sex, socioeconomic status, comorbidities, duration of symptoms, previous similar surgery, general health status, EQ-5D). Secondary outcomes were the risk-adjusted proportions of patients reporting any complications and rating their operation as excellent, very good, or good.

Risk-adjustment of outcomes was conducted using linear regression (logistic regression for post-operative complication incidence and overall operation success) with pre-operative PROM scores and other patient characteristics (age, sex, general health status, duration of symptoms, number of comorbidities, Index of Multiple Deprivation, previous similar surgery) being potential risk factors.

To account for possible clustering of patient outcomes within hospitals, we used robust standard errors. All P values are 2-sided and P values lower than 0.05 were considered a statistically significant result. Stata software was used for all calculations [24].

## Results

### Recruitment and response rates

During the recruitment period, 769 patients were recruited in ISTCs and 1895 in NHS providers. Data on the number of eligible patients were not obtained in 2 ISTCs and 3 NHS centres. However, in the other 4 ISTCs and 17 NHS centres, the proportion of eligible patients recruited in the ISTCs was higher: cataract surgery 72% v 66%; hernia repair 62% v 50%; varicose vein surgery 84% v 66%; hip replacement 98% v 54%; and knee replacement 100% v 56%.

There was little difference in post-operative response rate between patients treated in ISTCs and NHS facilities: cataract surgery 84% v 86%; hernia repair 70% v 77%; varicose vein surgery 68% v 74%; hip replacement 77% v 88%; and knee replacement 85% v 88%. In both treatment sectors and across all five procedures a comparison of the pre-operative disease-specific PROM scores of those who did and did not return a post-operative questionnaire revealed no statistically significant differences.

### Case-mix

Generally, patients undergoing day surgery in ISTCs were healthier and had a less severe primary condition than those in NHS facilities (Table 1): cataract patients were less likely to be in poor/fair health or to suffer any comorbidity, and their visual function was better; hernia patients were younger, had less comorbidity, had a shorter duration of symptoms, and were less likely to have undergone hernia repair before; and varicose vein patients were younger and were less likely to be in poor/fair health.

**Table 1:** Pre-operative characteristics of day surgery patients

Patient characteristic	Cataract surgery		Hernia repair		Varicose vein surgery	
	NHS (N = 590)	ISTC (N = 276)	NHS (N = 422)	ISTC (N = 69)	NHS (N = 269)	ISTC (N = 53)
	
	Mean (SD)	Mean (SD)	Mean (SD)	Mean (SD)	Mean (SD)	Mean (SD)
	
Age (years)	73.7 (10.6)	74.6 (10.0)	53.4 (18.0)	48.7 (14.1)	46.6 (13.5)	42.2 (16.5)
Duration symptoms (years)	2.8 (3.7)	2.6 (3.0)	2.8 (5.6)	1.6 (1.9)	13.6 (11.5)	12.8 (8.6)
Deprivation score	20.3 (14.1)	16.5 (9.3)	18.6 (15.5)	33.2 (18.9)	20.1 (14.8)	33.4 (21.7)
EQ-5D score	0.80 (0.24)	0.84 (0.22)	0.78 (0.18)	0.76 (0.19)	0.76 (0.19)	0.78 (0.16)
Disease-specific score	81.8 (17.9)	84.7 (15.9)	47.2 (9.2)	46.6 (9.2)	16.9 (8.3)	15.8 (8.4)
	
	N (%)	N (%)	N (%)	N (%)	N (%)	N (%)
	
Female	340 (57.7)	156 (56.5)	47 (11.1)	3 (4.3)	178 (66.2)	33 (62.3)
Any comorbidity	465 (78.8)	197 (71.4)	207 (49.1)	27 (39.1)	160 (59.5)	25 (57.2)
Previous similar surgery	202 (34.5)	90 (32.7)	84 (20.2)	9 (13.4)	86 (32.7)	15 (28.9)
Poor or fair health	131 (22.3)	45 (16.4)	34 (8.1)	4 (5.8)	27 (10.2)	3 (5.7)

Similar differences were observed for those undergoing joint replacement (Table 2): patients treated in ISTCs were less likely to be in poor/fair health or to suffer any comorbidity, their primary condition was less severe and, for hips, they were less likely to have undergone previous similar surgery.

**Table 2:** Pre-operative characteristics of joint replacement patients

Patient characteristic	Hip replacement		Knee replacement	
	NHS (N = 291)	ISTC (N = 184)	NHS (N = 323)	ISTC (N = 187)
	
	Mean (SD)	Mean (SD)	Mean (SD)	Mean (SD)
	
Age (years)	66.2 (14.6)	66.8 (14.1)	66.2 (16.5)	66.7 (12.2)
Duration symptoms (years)	3.9 (5.4)	3.7 (3.9)	8.1 (10.1)	8.6 (9.3)
Deprivation score	22.4 (16.8)	20.3 (13.3)	25.2 (17.4)	19.0 (12.6)
EQ-5D score	0.31 (0.31)	0.35 (0.32)	0.36 (0.32)	0.45 (0.30)
Disease-specific score	44.0 (7.7)	42.1 (8.4)	42.3 (7.5)	39.8 (8.0)
	
	N (%)	N (%)	N (%)	N (%)
	
Female	157 (54.1)	108 (58.7)	167 (52.0)	96 (51.6)
Any comorbidity	251 (86.2)	148 (80.4)	280 (86.7)	151 (80.7)
Previous similar surgery	79 (27.3)	34 (18.6)	168 (53.3)	78 (42.6)
Poor or fair health	62 (21.5)	27 (14.9)	68 (21.7)	21 (11.5)

### Patient-reported outcomes

For all procedures, except hernia repair, post-operative PROM scores in ISTC patients indicated better outcomes than those treated by NHS providers (Table 3). However, when adjustment was made for pre-operative characteristics statistically significant differences persisted for cataract surgery and hip replacement, both favouring patients treated at ISTCs: cataract surgery patients achieved a significantly better outcome on the VF14 (2.6 points on a 100-point scale, p = 0.005) and the EQ-5D (0.03 points on a 0 to 1 scale, p = 0.01); hip replacement patients on the OHS (2.4 points on a 70-point scale, p = 0.03) and the EQ-5D (0.06 points, p = 0.03).

**Table 3:** Unadjusted and adjusted post-operative PROM scores by operation

PROMs	NHS	ISTC	Adjusted difference*	95% CI	P-value
				
	Mean (SD)	Mean (SD)			
EQ-5D					
Cataract	0.77 (0.28)	0.84 (0.23)	0.03	0.009 to 0.06	0.01
Hernia	0.85 (0.21)	0.84 (0.22)	-0.02	-0.05 to 0.002	0.07
Varicose vein	0.87 (0.20)	0.89 (0.19)	0.005	-0.03 to 0.04	0.8
Hip	0.72 (0.26)	0.82 (0.20)	0.06	0.005 to 0.11	0.03
Knee	0.70 (0.26)	0.76 (0.22)	0.03	-0.02 to 0.08	0.2
Disease-specific					
Cataract (VF14)	92.3 (15.2)	96.2 (7.8)	2.6	0.97 to 4.2	0.005
Hernia (PCS)	50.1 (9.6)	51.8 (10.5)	1.2	-0.52 to 2.9	0.2
Varicose vein (AVVQ)	10.3 (9.0)	8.6 (7.9)	1.3	-3.1 to 0.49	0.5
Hip (OHS)	23.9 (9.9)	20.5 (7.6)	2.4	-4.4 to -0.36	0.03
Knee (OKS)	27.0 (9.3)	24.2 (8.8)	0.86	-3.7 to 2.0	0.5

*positive adjusted differences indicate better post-operative scores for ISTC patients

Fewer patients treated in ISTCs reported a post-operative complication than patients treated in NHS facilities, even after adjusting for pre-operative risk factors (Table 4). These differences were statistically significant for cataract surgery, hernia repair and knee replacement. Consideration of each of the four types of complication separately revealed that all four were reported less often by patients undergoing these procedures in ISTCs (Table 5).

**Table 4:** Proportion of patients reporting at least one complication by operation

Surgery type	NHS	ISTC	Adjusted odds ratio	95% CI	P-value
				
	N (%)	N (%)			
Cataract surgery	58 (11.5)	9 (3.9)	0.32	0.14 to 0.70	0.004
Hernia repair	79 (24.4)	8 (16.7)	0.46	0.32 to 0.66	<0.001
Varicose vein	83 (41.5)	13 (36.1)	0.75	0.42 to 1.3	0.3
Hip replacement	87 (33.9)	41 (29.1)	0.87	0.52 to 1.5	0.6
Knee replacement	112 (39.6)	39 (24.5)	0.43	0.27 to 0.69	<0.001

**Table 5:** Proportion of patients reporting complications by operation

Operation	NHS	ISTC	Adjusted odds ratio	95% CI	P-value
				
	N (%)	N (%)			
Cataract					
Bleeding	17 (3.4)	5 (2.2)	0.74	0.37 to 1.5	0.4
Wound infection	18 (3.6)	2 (0.9)	0.13	0.02 to 1.0	0.05
Urinary problems	12 (2.4)	1 (0.4)	0.26	0.03 to 2.5	0.2
Adverse drug reaction	19 (3.8)	4 (1.7)	0.53	0.19 to 1.5	0.2
Hernia repair					
Bleeding	19 (5.9)	2 (4.2)	0.80	0.35 to 1.8	0.6
Wound infection	35 (10.8)	4 (8.3)	0.58	0.33 to 1.0	0.06
Urinary problems	20 (6.2)	3 (6.2)	0.82	0.43 to 1.5	0.5
Adverse drug reaction	21 (6.5)	1 (2.1)	0.16	0.10 to 0.27	<0.001
Varicose vein surgery					
Bleeding	51 (25.5)	8 (22.2)	0.71	0.30 to 1.7	0.4
Wound infection	50 (25.0)	9 (25.0)	1.2	0.74 to 2.0	0.4
Urinary problems	7 (3.5)	1 (2.8)	1.2	0.46 to 3.3	0.7
Adverse drug reaction	10 (5.0)	0 (0)	1.0	0.94 to 1.0	0.7
Hip replacment					
Bleeding	27 (10.5)	10 (7.1)	0.88	0.47 to 1.7	0.7
Wound infection	27 (10.5)	15 (10.6)	1.2	0.58 to 2.6	0.6
Urinary problems	33 (12.8)	12 (8.5)	0.67	0.35 to 1.3	0.2
Adverse drug reaction	29 (11.3)	15 (10.6)	0.84	0.39 to 1.8	0.6
Knee replacement					
Bleeding	25 (8.8)	7 (4.4)	0.45	0.14 to 1.4	0.2
Wound infection	42 (14.8)	14 (8.8)	0.50	0.28 to 0.90	0.02
Urinary problems	42 (14.8)	13 (8.2)	0.51	0.29 to 0.88	0.02
Adverse drug reaction	39 (13.8)	16 (10.1)	0.65	0.43 to 0.97	0.04

Most patients described the result of their operation as a success (i.e. excellent, very good or good) both in ISTCs and NHS facilities: cataract surgery 97% v 91%; hernia surgery 98% v 94%; varicose vein surgery 71% v 86%; hip replacement 98% v 92%; and knee replacement 85% v 87%. Following risk adjustment patients undergoing varicose vein surgery in an ISTC were less likely to describe their operation as a success (Adjusted Odds Ratio 0.38; 95% CI 0.15–0.90; p = 0.03) while those undergoing hernia surgery were more likely to declare it a success (Adjusted Odds Ratio 3.2; 95% CI 1.2–8.1; p = 0.02).

## Discussion

### Findings

In line with their contracts, ISTCs tend to treat healthier patients with less severe primary conditions. Having adjusted for such differences in case-mix, patients reported similar improvements in function and health-related quality of life following hernia repair, varicose vein surgery and knee replacement in ISTCs and NHS providers. Improvements following cataract surgery and hip replacement were slightly greater in patients treated in ISTCs. There was also a lower incidence of complications reported by ISTC patients undergoing cataract, hernia and knee surgery.

### Limitations

There are four reasons for caution in interpreting these comparisons between ISTCs and NHS providers. First, although a high proportion of eligible patients were recruited in the ISTCs, the small number of ISTCs that participated limits the generalisability of the results. While patients undergoing joint replacement were recruited from four ISTCs, only one ISTC was involved for hernia and varicose vein surgery. Second, a lower proportion of patients were recruited in NHS centres than in ISTCs. It is possible that this difference could have introduced a bias. Third, the risk adjustment models had only poor predictive power (area under ROC curve about 0.6). It is probable that the addition of clinician-reported data would improve the predictive power of our models. Fourth, there may have been some reporting bias. If ISTC patients were more satisfied with their experience, as has been reported [14], they may have been less critical of their outcome.

While not undermining comparisons between types of provider, the validity and reliability of the absolute values reported could be challenged. Although the instruments used were the best available in psychometric terms, the validity of at least one (the VF14) has been challenged by clinicians [25], though it is unclear how justified such criticism is. Another concern is the validity of patients' reporting of complications: patients may wrongly attribute a problem to the operation and/or they may interpret normal post-operative recovery, such as some wound discomfort, to be a 'complication'. Despite these concerns, there is evidence that the question has sufficient construct validity for comparative purposes: reports of bleeding and wound problems were much higher following varicose vein surgery (around 25%) than after cataract surgery (around 3%) and the incidence of urinary problems was higher after joint replacement (12%) than after hernia repair (6%) or varicose vein or cataract surgery (2–3%).

### Implications

These, the first quantitative data to cast light on the effectiveness of surgery in ISTCs, suggest there is no widespread problem with poor quality care. Indeed, the lower incidence of patient-reported complications following treatment in an ISTC is reassuring. These findings do not refute previous reports of individual instances of serious lapses in the quality care in some ISTCs, they simply suggest that such failures are uncommon. Inevitably, there is no information yet available as to the long-term outcomes.

Although these findings provide some systematic evidence about the effectiveness and safety of ISTCs, the conclusions have to be tentative given the relatively small sample of providers and patients. A larger sample would enable the generalisability of these findings to be tested, provide greater statistical power, and allow more robust risk-adjustment models to take into account case-mix differences. In addition, the inclusion of clinician-reported outcomes would allow an assessment of the effect of surgery on impairments (such as visual acuity and extent of joint movement) and enable a clinical view of the incidence of complications to be obtained. An audit of hip and knee replacements in all ISTCs and representative sample of NHS providers is due to start soon.

## Conclusion

The case-mix of patients treated in ISTCs differs from that in NHS providers, in line with the intention of the contracts. Caution is needed in interpreting the observation that patients treated in ISTCs reported slightly better outcomes as very few ISTCs participated, case-mix adjustment might have been insufficient, and patients' reports might have been biased as they were more likely to be satisfied with the way they were treated.

## Competing interests

The author(s) declare that they have no competing interests.

## Authors' contributions

JB, NB and JvdM conceived and designed the study; LJ and LC managed the data collection; JL analysed the data; NB drafted the paper and all authors contributed to the final draft. NB acts as guarantor.

## Pre-publication history

The pre-publication history for this paper can be accessed here:


